# Liver Pathology in Rats Treated with Newcastle Disease Virus Strains AF2240 and V4-UPM

**DOI:** 10.31557/APJCP.2019.20.10.3071

**Published:** 2019

**Authors:** Rowa Mohammed Assayaghi, Aied Mohammed Alabsi, Gumballi Swethadri, Abdul Manaf Ali

**Affiliations:** 1 *Department of Microbiology and Immunology, Faculty of Medicine and Health Sciences, Sana’a University, Sana'a, Yemen,*; 2 *Faculty of dentistry, MAHSA University, Jenjarom, *; 3 *Department of Pathology, Faculty of Medicine,*; 4 *Faculty of Bioresources and Food Industry, Universiti Sultan Zainal Abidin,Kota Campus, Terengganu, Malaysia.*

**Keywords:** Liver, oncolytic virus, NDV, NDV AF2240, NDV V4, UPM, Azoxymethane, 5-FU

## Abstract

**Background:**

Treatment of cancer with chemo-radiotherapy causes severe side effects due to cytotoxic effects towards normal tissues which often results in morbidity. Therefore, developing anticancer agents which can selectively target the cancer cells and cause less side effects are the main objectives of the new therapeutic strategies for treatment advanced or metastatic cancers. Newcastle disease virus strains AF2240 and V4-UPM were shown to be cytolytic against various cancer cells in-vitro and very effective as antileukemicagents.

**Methods:**

45 rats at 6 weeks of age, were randomly assigned to nine groups with 5 rats in each group, both azoxymethane (AOM) and 5-Fluorouracil (5-FU) were given to rats according to the body weight. NDV virus strains (AF2240 and V4-UPM) doses were determined to rats according to CD50 resulted from MTT assay. After 8 doses of NDV strians and 5-FU, tissue sections preparations and histopathological study of rats’ organs were done.

**Results:**

In this article morphological changes of rats’ organs, especially in livers, after treatment with a colon carcinogen (azoxymethane) and Newcastle disease virus strains have been recorded. We observed liver damage caused by AOM evidenced by morphological changes and enzymatic elevation were protected by the oncolytic viruses sections. Also we found that combination treatment NDV with 5-FU had greater antitumor efficacy than treatment with NDV or 5-FU alone.

**Conclusion:**

We noted morphological changes in liver and other rats’ organs due to a chemical carcinogen and their protection by NDV AF2240 and NDV V4-UPM seems to be most protective.

## Introduction

Newcastle disease virus (NDV) is a member of the genus Avulavirus in the family Paramyxoviridae (Alexander etal., 2012). NDV is classified as a virus with inherent oncolytic properties which anti-cancer effect was first reported by Cassel and Garret in 1965 (Omar et al., 2003). Since then NDV has been investigated for its anti-cancer effects and scientists are interested in NDV because it can replicate up to 10,000 times better in human neoplastically transformed cells than in most normal human cells (Nelson, 1999; Pecora, 2002). Two Malaysian NDV strains that have antineoplastic properties were discovered ; AF2240 and V4-UPM (Tan etal., 1995; Othman etal., 2010). These two strains are currently being tested as anticancer agents in vivo and in vitro against different types of cancers such as leukemia, brain and breast cancer (Zulkifli et al., 2009; Alabsi et al., 2012; Ghrici et al., 2013). Azoxymethane (AOM) is an agent widely used in experimental models of colorectal carcinogenesis in rodents, it is highly specific indirect colorectal carcinogens that induce the initiation and promotion steps of colorectal carcinogenesis yielding colorectal tumors lesions in a dose-dependent manner in rats, mice and hamsters (Kobaek-Larsen, 2002).

## Materials and Methods

*Animal experiment protocol*

The experimental protocol was approved by Animal Ethics Committee, University Sultan Zainal Abdin (UniSZAAEC); with an ethic no. (UniSZA/AEC 02/09/03). At 6 weeks of age, 45 rats were randomly assigned to nine groups with 5 rats in each group so that there was no difference in body weight among the groups. Both azoxymethane (AOM) (Sigma, Aldrich) and 5-Fluorouracil (5-FU) (Hospira, Australia) were given to rats according to the body weight, AOM dose was 15 mg/kg and FU dose was 12 mg/kg. Hundred mg of AOM ampoule was diluted in 10 mL sterile normal saline (0.9%) and stored at -20°C until used. NDV virus strains (AF2240 and V4-UPM) doses were determined according to CD50 resulted from MTT assay. Each low and high dose of NDV strains was diluted in 500 µL sterile normal saline (0.9%) before injection.

The 9 groups of rats were as follows; a negative control (NC) served as normal control and were administrated with normal saline only, a positive control (PC) which rats were administrated subcutaneous injections of 15 mg/kg AOM and not treated with NDV, group III which received low doses of NDV AF2240 (AFLD), group IV which was given low doses of NDV V4-UPM (V4LD), group V that received high doses of NDV AF2240 (AFHD), group VI which received high doses of NDV V4-UPM (V4HD), group VII received both NDV AF2240 and 5-Fluorouracil (FU) (AFFU), VIII received both NDV V4-UPM and 5-Fluorouracil (FU) (V4FU) and the last group that treated with 5-Fluorouracil (FU).

All rats (except the negative group) were then administered a subcutaneous injection of 15 mg/kg AOM with a second dose of 15 mg/kg AOM administered one week later. Four weeks post AOM treatment; rats administrated intraperitoneal injections of 8 doses of NDV and 5-FU. 

Doses were administrated once daily for 4 successive days; then if no toxicity was observed doses were given on the 6^th^, 8^th^, 10^th^, and 12^th^ day (No therapy was given on days 5, 7, 9, or 11). A week later, those rats were fasted for 12h overnight. After euthanasia, rats were sacrificed and then organs were removed and kept in 10% neutral formalin until used. 

*Tissue sections preparations and histopathological study of rats’ organs*

For histological observation, fresh tissue pieces of colon, liver, kidney and spleen from the 9 groups of rats were taken. All samples were placed in tissue embedding cassettes and fixed 10% buffered formaldehyde solution for minimum 48h. Following fixation, the specimens were dehydrated in ascending grades of alcohol by Automatic Tissue Processer (Leica ASP 300S, Germany) for 18-24 h. Tissues were embedded in paraffin wax and blocks were made by using Tissue Embedding Center (Leica EG 1160, Germany). Sections were made 5–7µm thick by Semi-Motorized Rotary Microtome (Leica, Germany) and were double stained with hematoxylin and eosins (HE) stain. All prepared histological sections were viewed in a light microscope equipped with an Olympus digital camera (Olympus, Japan) (Bird and Lafave, 1995). Histopathological study was done by a histopathologist (3rd author) and the classification of colon tissue sections was based on three types of dysplasia; Mild, Moderate and Severe dysplasia (Qing et al., 2008).

The discussion on production of dysplasia, prevention by newcastle virus is presented in an article by the authors elsewhere. This article pertains to organ changes in liver, kidneys and spleens. Histological sections were studied for signs of abnormalities or cancer metastasis. However, when reviewing liver sections it was noted that there were morphological changes which the authors felt that they should be recorded. These rats (except negative control) were treated with azoxymethane to induce dysplasia in colon and to observe effect of NDV strains on these dysplasia, incidentally these two agents did cause some morhological changes especially in livers.

*Statistical analysis*

The statistical significance was assessed using one analysis of variance (ANOVA). All values were expressed as mean ± SE and a value of P ≤ 0.05 was considered significant as compared to the respective control groups using IBM SPSS 20 statistical package for Microsoft Windows (SPSS Inc. Chicago, IL, USA).

## Results

*The morphological changes observed in liver sections*

Positive control rats liver hematoxylin and eosins (HE) stained sections reveals the least number of hepatocytes among all the groups of rats suggesting AOM has deleterious effect on liver cells. Supported also by three times elevation in aspartate transaminase and two times elevetion in alanine transaminase according to blood tests profile (not shown) in the same group.

**Table 1:** Morphological Changes Observed in Liver Hematoxylin and Eosins (HE) Stained Sections

Rat group	Hepatocyte/lobule	Kupfer cells	Portal triads	apoptosis	Fat cyst	necrosis	Eosinophilia hepatocyte	P*
NC	421	20	4	1	0	-	-	
PC	210	16	10	0	12	-	-	
AFLD	360	55	4	0	0	+	+	
AFHD	240	160	4	0	0	-	+	
AFFU	280	nil	6	0	0	-	++	<0.001
V4LD	300	23	4	0	0	-	+	
V4HD	280	14	4	0	2	-	-	
V4FU	300	40	4	0	0	-	-	
FU	360	29	5	0	0	+	-	

* P value ≤ 0.05 is significant; P value > 0.05 is non-significant; (+), mild; (++), moderate; (-), not seen; NC, Negative control group; PC, Positive control (non-treated) group; AFLD, Rats were treated with low dose of NDV strain AF 2240; AFHD, Rats were treated with high dose of NDV strain AF 2240; AFFU, Rats were treated with NDV strain AF2240 + 5-Fluorouracil; V4LD, Rats were treated with low dose of NDV strain V4-UPM; V4HD, Rats were treated with high dose of NDV strain V4-UPM; V4FU, Rats were treated with NDV strain V4-UPM + 5-Fluorouracil; FU, Rats were treated with 5-Fluorouracil

**Figure 1:**
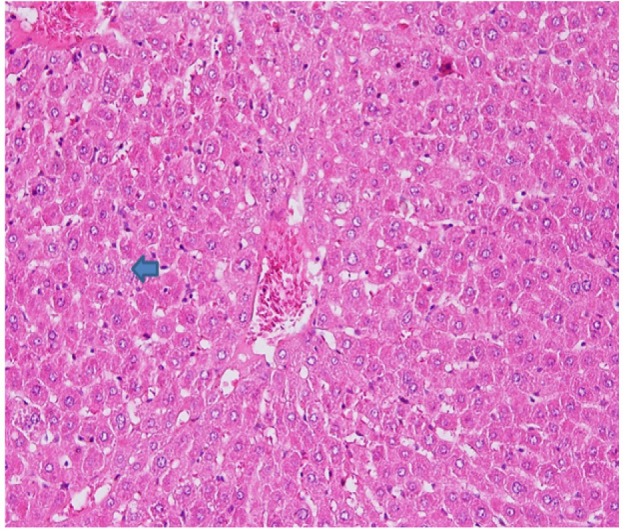
Rat Liver Hematoxylin and Eosins (HE) Stained Section: Kupfer Changes Hyperplasia with Fatty Cells (Arrow)

**Figure 2.**
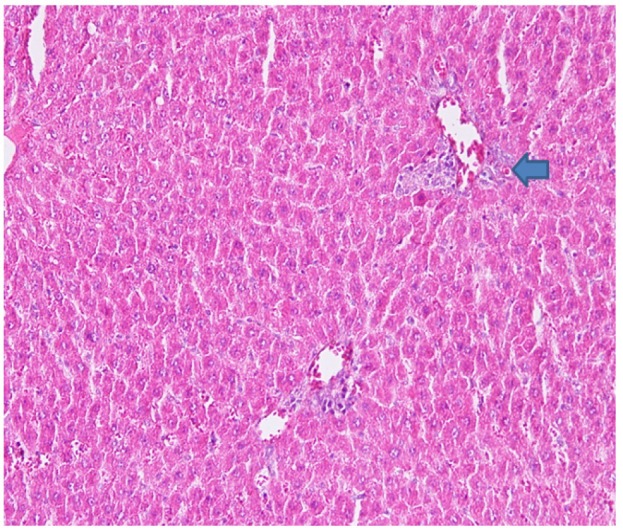
Rat Liver Hematoxylin and Eosins (HE) stained section: Eosinophilia hepatocytes (councilman body) (arrow)

**Figure 3:**
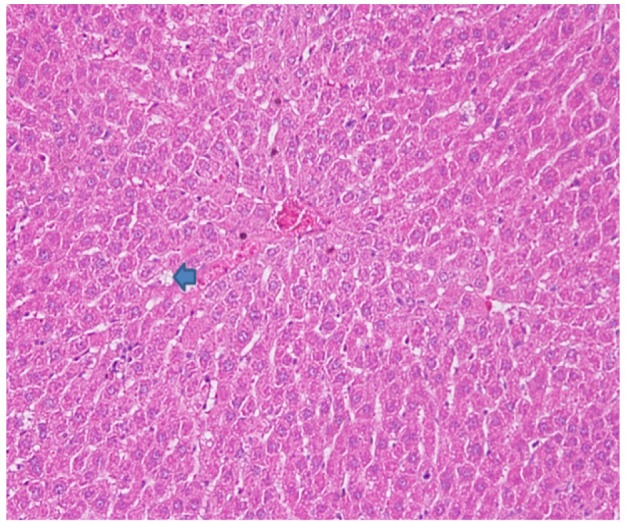
Rat Liver Hematoxylin and Eosins (HE) Stained Section: Fatty Changes (Arrow)

**Figure 4:**
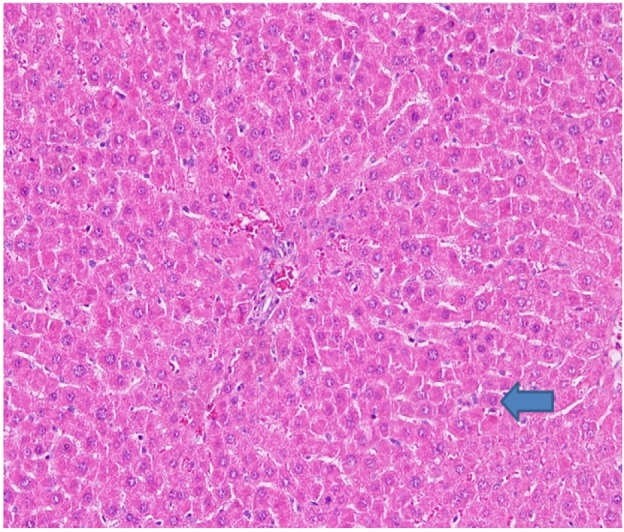
Rat Liver Hematoxylin and Eosins (HE): Eosinophilia Hepatocytes with Some Necrotic Cells (Arrow)

Kupfer cells are also decreased except in AFHD group (Figure 1). Increase in portal triads can be attributed to decrease in size of lobule bringing together closeness of portal tracts. 12 fat cysts also evidence of greater damage to liver suggesting that AOM in the doses used to induce dysplastic changes induce above changes in liver (Figure 1 and Figure 3). It was interesting to observe liver damage caused by AOM evidenced by morphological changes and enzymatic elevation were protected by the oncolytic viruses. There was significant difference (P ≤ 0.05) among the rat groups compared to positive (non-treated) group. 

In this aspect low and high doses of NDV AF2240 were less effective in preventing damage but low and high doses of NDV V4-UPM were better, however, AFLD, AFHD,V4LD all showed eosinophilia of hepatocyte cytoplasm which is a morphological evidence of hepatocyte damage (Figure 2 and Figure 4).

In V4HD rat group showed no enzymatic and morphological changes so we may assume it is most protective, also it is well known that cytoplasmic eosinophilia is caused by ischemic canges due to ATP depletion, the action may be drug induced here. The combination of NDV V4-UPM and 5-FU shows more protective than NDV AF2240 and 5-FU. The damage (necrosis) observed in AFLD and FU groups are suggesting that combination of low dose NDV AF2240 and 5-FU are not able to protect the damage caused by AOM, but low dose NDV V4-UPM is better than 5-FU in this aspect.

In AFHD rats showed kupfer cell hyperplasia it may have irrtating effect/reactive change on kupfer cells in this dose. High dose NDV AF2240 has more irritative effect than low dose NDV AF2240. Necrosis is found only in FU and AFLD rat groups (Figure 4) which is due to cytotoxic effect.

NDV AF2240 may have different effect based on dosage former acting on kupfer cells and latter on hepatocytes. AFHD group has characteristc microvescicular fat change in cytoplasm (Table 1).

*The morphological changes observed in Kideny and spleen sections*

Histopathological study of kidney HE stained sections revealed no metastasis. There were no evidence of glomerular damage except for the finding of 14 glomeruli per one high power field in PC group as against 18 in all other samples was statistically non-significant, so it is concluded that there were no significant kidney changes.

Sections from spleen HE stained sections showed no metastasis, there were occational reactive hyperpalsia, this is not considered significant in lymphoid tissues.

## Discussion

Many reports showed the possibility of NDV as a therapeutic agent in cancer treatment, from studies both in animal models and in human clinical trials which showed favorable results (Omar et al., 2003; Krishnamurthy et al., 2006).

In this study, we examined rats HE stained sections of liver, kidney and spleen. However, the most significant cell changes were found in livers compared treated with AOM to those treated with NDV strains. 

Positive control rats liver HE stained sections reveals the least number of hepatocytes among all the groups of rats suggesting AOM has deleterious effect on liver cells (Kobaek-Larsen, 2002). Supported also by three times elevation in aspartate transaminase and two times elevetion in alanine transaminase according to blood tests profile. Kupfer cells are also decreased except in AFHD group. Increase in portal triads can be attributed to decrease in size of lobule bringing together closeness of portal tracts. 12 fat cysts also evidence of greater damage to liver suggesting that AOM in the doses used to induce dysplastic changes induce changes in liver. We observed liver damages caused by AOM evidenced by morphological changes and enzymatic elevation were protected by the oncolytic viruses. These results were also shown in previuos studies which mentioned that NDV AF2240 and NDV V4-UPM have cytolytic effect on different types of cancer cells, also no toxicity was recorded in these studies of even high doses of NDV strains (Zulkifli et al., 2009; Alabsi et al., 2012; Ghrici et al., 2013). 

This study confirmed that combination of NDV and 5-FU had greater antitumor efficacy than NDV or 5-FU alone. These results were in agreement with a previous study which showed the synergistic effect of combination of NDV strain LaSota with Methotrexate on three types of cancer cell lines (Al-Shammari and Yaseen, 2012). Moreover, NDV combination with vinorelbine and carboplatin was tested on non-small cell lung cancer (NSCLC) and the results showed significantly enhanced efficacy, reduce toxicity, synergistic anticancer chemotherapy (Ying, 2011). The mechanisms of synergistic activity in the combination of 5-FU with NDV is thought to be that maybe NDV enhance the antitumor activity of 5-FU by increasing cellular sensitivity to it then enhance induction of cells apoptosis which is previously proved to be induced by NDV (Fabian et al., 2001; Washburn and Schirrmacher, 2002; Al-Shammari and Yaseen, 2012).

In conclusion, we noted morphological changes in liver due to a chemical carcinogen and their protection by NDV AF2240 and NDV V4-UPM seems to be most protective. The results also proved that combination of NDV and 5-FU had greater antitumor efficacy than treatment with NDV or 5-FU alone. All these results confirmed that NDV strains are good antitumor agents in-vivo and can be used in preclinical studies.

These subjective observations in our small number of samples need to be confirmed by larger cohort of animals. The number of samples is small and the conclusions derived are subjective so further studies with large number of samples and morphometry is suggested.
